# Duodenogastric Intussusception in a 14-Week-Old Infant with Donohue Syndrome: Case Study

**DOI:** 10.1155/2023/7799234

**Published:** 2023-10-18

**Authors:** Corina Ramona Nicolescu, Clara Cremillieux, Jean-Louis Stephan

**Affiliations:** Department of Pediatrics, Centre Hospitalier Universitaire, Saint-Etienne, France

## Abstract

Donohue syndrome (DS) is a rare recessively inherited disorder characterized by severe insulin resistance caused by genetic defects affecting the insulin receptor. The classical clinical characteristics include severe intrauterine growth restriction, craniofacial dysmorphic features, body and skin features, and soft tissue overgrowth. Postnatal growth retardation, cardiac, gastrointestinal, and renal complications, and infection susceptibility develop within the first few months of life, leading to a short life expectancy (<2 years). The classical metabolic abnormalities vary from fasting hypoglycemia to postprandial hyperglycemia with severe hyperinsulinemia. We present the case of a 14-week-old infant with DS who developed cardiac, renal, hepatic, pancreatic, and gastrointestinal features, all of them previously reported in infants with DS. The gastrointestinal features started during the first week of life and included abdominal distension, feeding difficulties, intermittent vomiting, and two episodes of intestinal obstruction. The diagnosis of duodenogastric intussusception was made, and this previously unreported complication tragically resulted in mortality. We discuss how basic mechanisms of cross-talk between insulin and insulin-growth factor 1 receptors could be linked to hyperinsulinemia and its associated comorbidities.

## 1. Introduction

Donohue syndrome (DS) is the most severe manifestation of insulin resistance (IR) and is caused by homozygous or compound heterozygous mutations in the insulin receptor (INSR) gene.

The neonatal clinical phenotype includes intrauterine growth restriction (IUGR), morphological aberrations, fasting hypoglycemia, postprandial hyperglycemia, hyperinsulinemia, and initial resistance to ketosis [1]. Postnatal growth retardation, cardiac, gastrointestinal, and renal complications, and infection susceptibility develop within the first few months of life.

Ovarian enlargement and juvenile granulosa cell tumors may be associated.

The prognosis is poor, with cardiac, gastrointestinal, and infectious morbidity and high mortality during the first years of life.

The main biochemical features are defined by abnormal glucose homeostasis, with hypoglycemia, postprandial hyperglycemia, and severe hyperinsulinemia, and are present soon after birth.

The most common pharmacological approach for overcoming tissue insulin resistance is the use of recombinant human insulin-like growth factor 1 (IGF_1_), although the complexity of its various tissue effects remains only partially understood.

We report the case of a 14-week-old infant with DS and rapid development of classical cardiac, renal, hepatic, and pancreatic complications. The gastrointestinal involvement presented initially as feeding difficulties, intermittent vomiting, and abdominal distension without organomegaly and worsened rapidly, culminating in a fatal duodenogastric intussusception, confirmed by radiological and histopathology studies.

With this report, we would like to raise awareness of the possibility of early severe gastrointestinal dysmotility in infants with DS.

## 2. Case Report

We describe the case of a male newborn, fifth child of a consanguineous Caucasian couple (first cousins), born at 31 weeks of gestation, weighing 950 g (<<−3 SD), and measuring 43 cm (−1 SD).

Severe intrauterine growth retardation was evident throughout pregnancy, and at birth, the newborn appeared markedly emaciated, with no subcutaneous fat, muscular hypotrophy, and dysmorphic features: elfin face, low-set ears, broad nasal tip, thick lips with broad mouth, macroglossia, generalized hypertrichosis, small thorax, distended abdomen without hepatosplenomegaly, and macrogenitosomia. Acanthosis nigricans was absent.

On the first day of life, the infant had hyperglycemia (12 mmol/L) with very high random insulin (932 mUI/L) and C peptide (3.46 nmol/L) levels. Complementary endocrine findings included low levels of insulin growth factor 1 (IGF_1_) (12 *µ*g/L) and insulin growth factor-binding protein 3 (IGFBP_3_) (0.35 mg/L) with a normal growth hormone level (26 mU/L) and an undetectable leptin level (<1 ng/mL). Echocardiography indicated a 3 mm ductus arteriosus with no other morphological abnormalities.

The constellation of intrauterine growth restriction, distinctive clinical features, and disrupted glucose homeostasis strongly suggested the diagnosis of Donohue syndrome (DS).

The patient's parents provided informed consent for molecular genetic analysis, which confirmed the diagnosis of DS through the detection of the homozygous variant c.1106T > A in exon 4 of the insulin receptor gene. This variant results in an amino acid substitution of Ile369 with Asn (I369N) within the insulin-binding domain. Both parents were heterozygous carriers of this missense variant, which was not previously reported.

To manage the disrupted glucose homeostasis, continuous subcutaneous administration of recombinant human IGF_1_ (rhIGF_1_) was initiated at 15 days of life with a progressively adjusted dosage from 60 to 500 *µ*g/kg/day, resulting in improved glycemic control (glucose level between 4 and 10 mmol/L) without hypoglycemia, normalized IGF_1_ and IFGBP_3_, and unchanged insulin levels.

The echocardiography follow-up revealed severe left ventricular hypertrophy at the age of 14 days and global myocardial hypertrophy at 11 weeks of age, with interventricular septal thickness at diastole of 8 mm, left ventricular outflow tract obstruction, and ventricular ejection fraction at 37%. Treatment with a *β*-blocker (propranolol) was proposed, but not immediately initiated.

Renal dysfunction developed at 2 weeks, with progressive array of electrolyte disturbances including hyponatremia, hypokalemia, hypophosphatemia, hypomagnesemia, hypercalciuria, and proteinuria. At 11 weeks, ultrasonography revealed nephrocalcinosis without renal hypertrophy.

Since birth, the neonate presented feeding difficulties, nonbilious vomiting, and unexplained abdominal distension.

At the age of 10 days, the neonate developed blood emesis with exacerbation of the abdominal distension, without hepatomegaly. The hepatic profile revealed elevated transaminase levels, prolonged prothrombin time, low serum albumin concentrations, and elevated total and conjugated bilirubin levels. Pancreatic exocrine dysfunction was suspected based on lipase levels and fecal elastase levels, although a confirmatory examination was not conducted (Table 1).

The baby was initially fed by parenteral and then enteral nutrition (by nasogastric tube), with an adequate caloric intake (130 kcal/kg/d) and low weight progression.

**Figure 1:**
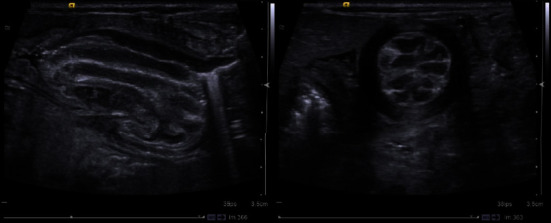
Longitudinal and transverse ultrasound views of the pylorus displaying intussusception.

**Figure 2:**
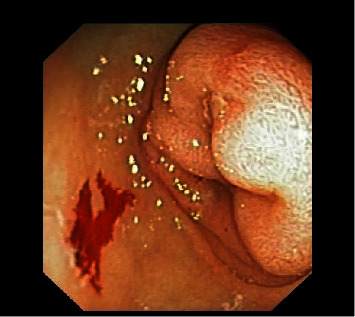
Duodenogastric intussusception on endoscopy.

At 11 weeks of age, the infant had a new episode of acute abdominal distension with vomiting. Radiological evaluation revealed significant gastric distension on plain film radiography and duodenogastric intussusception on ultrasonography (Figure 1). Upper endoscopy confirmed the duodenogastric intussusception (Figure 2) and revealed a hypertrophic, pale, and edematous gastric mucosa with enlarged folds. Attempt to reduce intussusception by endoscopy was unsuccessful. Histological examination revealed an unremarkable duodenal mucosa with diffuse thickening of the antral gastric mucosa, crypt hypertrophy, and lymphoplasmacytic infiltration. Immunohistochemical analysis was negative for *Helicobacter pylori* and *Cytomegalovirus* infections.

To overcome the partial gastric outlet obstruction and optimize the enteral feeding, gastrostomy and gastrojejunal tubes were placed, but there was little or no clinical improvement. Surgery was discussed, but due to fragile cardiac condition of the infant, temporizing surgical management was considered.

The infant's clinical evolution did not improve, and he required increasing supplemental oxygen and developed more frequent episodes of tachyarrhythmia and hypertension. Continuous positive airway pressure support was provided.

At the age of 14 weeks, the infant's clinical status deteriorated, with severe abdominal distension and incoercible vomiting, suggestive of new episode of intestinal intussusception with associated cardiopulmonary distress, ultimately leading to death. Autopsy was not performed (parental decision).

## 3. Discussion

Our patient presented with common clinical and biological features of DS. Facial, skin, and bodily features, overgrowth of soft tissues, and organ dysfunction (renal, hepatic, pancreatic, and gastrointestinal) were present at birth or developed early during the neonatal period. His initial biological picture was also classic, with severe hyperinsulinemia, postprandial hyperglycemia, and low growth hormone level.

We hypothesized that the variant c.1106T > A in exon 4 of the insulin receptor gene, found in our patient and his parents, likely alters insulin-binding kinetics, although this has not yet been demonstrated. Several analytical criteria support the pathogenic nature of this variant, including its absence in the general population (gnomAD) and bioinformatics prediction indicating potential pathogenicity.

Hypertrophic cardiomyopathy [2], renal enlargement and dysfunction (electrolyte abnormalities) [3, 4], genital enlargement with biochemical abnormalities [5, 6], and gastrointestinal functional features [7, 8] were previously reported in children with DS.

Gastrointestinal picture of DS includes intrauterine growth restriction, poor postnatal weight gain, and abdominal distension. Recently, additional digestive manifestations, such as intrinsic gastrointestinal dysmotility, hepatic dysfunction, and pancreatic exocrine insufficiency, were reported [8].

Our patient's gastrointestinal features and evolution, including early feeding difficulties, repeated vomiting of unclear cause, and progressive to permanent abdominal distension with 2 episodes of exacerbation (acutely distended abdomen with massive vomiting), culminated in the diagnosis of duodenogastric intussusception (ultrasound and endoscopic diagnosis). This entity was not previously reported in patients with DS, and its clinical, radiological, and histological characteristics, as found in our patient, could add important insights into the complex gastrointestinal picture of DS. Ultrasonography remains a valuable imaging study option for gastrointestinal emergency diagnosis in infants [9, 10].

The severity of this gastrointestinal complication in the context of rapid progressive hypertrophic cardiomyopathy with associated compromised hemodynamic stability contributed to fatal evolution of our patient.

DS is an extremely rare form of insulin resistance in the pediatric population, and the main pathophysiological mechanism of this glucose homeostasis abnormality is a result of genetic defects in the INSR.

Insulin and IGF_1_ act via structurally and functionally similar tyrosine kinase receptors and have similar physiological effects on vital metabolic activities and developmental processes (cellular growth, proliferation, differentiation, apoptosis, and survival) [11, 12]. Receptors' structural and functional homology may become significant only at supraphysiological concentrations of insulin and IGF_1_.

The molecular anomalies of the INSR, the insulin mimetic on the IGF_1_ receptors (IGF_1_R) leading to their pathological hyperactivation, and IGF_1_R tissue expression contribute to the phenotypic and biological aberrations in DS.

IGF_1_R is lowly expressed in insulin-responsive tissues, such as adipocytes [13], which contributes to reduced subcutaneous fat in DS.

IGF_1_R is expressed in the cardiac tissue, and hyperinsulinemia-mediated IGF_1_R signaling is implicated in cardiomyocyte hypertrophy [14].

IGF_1_ is involved in the tubular handling of sodium, water, calcium, and phosphate and regulates tubular gluconeogenesis [3]. Insulin signaling through the IGF_1_R may contribute to renal enlargement and electrolyte imbalance observed in patients with DS.

The insulin/IGF_1_ system is vital for mammalian sexual development and reproduction [15]. In DS, the overactivation of this system results in genital enlargement and biochemical abnormalities (elevated estradiol and testosterone levels).

Activation of the IGF_1_R by insulin, along with low circulating growth hormone levels, may serve as a protective mechanism against ketoacidosis in patients with DS [1].

The gastrointestinal tract is the primary target of IGF_1_ action, which stimulates the proliferation of intestinal epithelial and muscle cells [16]. In mice, IGF_1_ overexpression has been shown to lead to a normal gastrointestinal epithelium, but an expanded submucosa with hyperplastic and hypertrophic muscularis propria [17].

Did our patient's tragic evolution (duodenogastric mucosal intussusception in the context of severe myocardial hypertrophy) reflect the natural progression of DS or was related to rhIGF_1_ treatment?

rhIGF_1_ therapy appears to improve glycemic control in most patients with DS, although there are theoretical concerns that rhIGF_1_ may worsen DS complications. Clinical data on the pharmacological effects of rhIGF_1_ (beneficial or deleterious) on hypertrophic cardiomyopathy development and progression in DS patients are limited. Additionally, abdominal distension was present in almost all reported DS cases, and the infants not treated with rhIGF_1_ died [18, 19]. Possible mechanisms involve IGF_1_ and insulin signal transduction inhibiting apoptosis and stimulating cellular proliferation and carcinogenesis [20].

## 4. Conclusion

Our observation sheds light on the complex phenotypic features of DS patients, with particular attention to the gastrointestinal manifestations. Although less prevalent and less diagnosed, they display significant variability in clinical presentation and severity and influence the patient survival. Accumulating clinical evidence may eventually lead to earlier diagnosis of DS gastrointestinal dysfunction and timely and appropriate intervention.

## Figures and Tables

**Table 1:** Patient's clinical and biochemical evolution.

Age	Clinical features	Laboratory data	Normal values
At birth	Intrauterine growth retardation	Glycemia 12	3.9–7 mmol/L

1 week	*Morphological characteristics* Elfin face, low-set ears, broad nasal tip, and thick lips with broad mouth Hypertrichosis Emaciation with adipose tissue and muscular hypotrophy Organomegaly (tongue and external genitalia) Distended abdomen Small chest No acanthosis nigricans	Insulin 972 C peptide 3.46 IGF_1_ 12 IGFBP_3_ 0.35 GH 26 Leptin <1	2–13 mU/L 0.37–1.47 nmol/L 18–156 *µ*g/L 0.7–1.4 mg/L <17 mU/L 2.45–8 ng/mL

2 weeks	*Gastrointestinal and nutritional findings* Poor weight gain Hepatic dysfunction Pancreatic deficiency Feeding difficulties Hematemesis Progressive abdominal distension	ALT 172 AST 232 Total bilirubin 105 Conjugated bilirubin 34	<50 IU/L 10–50 IU/L <17.8 *µ*mol/L <3.4 *µ*mol/L
	*Renal findings* Electrolyte abnormalities Nephrocalcinosis	Prothrombin time 17 (51%) Albumin 24 Lipase 14	12–13 s 29–44 g/L 151–238 U/L
	*Cardiac findings* Severe left ventricular Hypertrophy	Fecal elastase 132 Na 132 K 2.7 Mg 0.55 Proteinuria 67 Urinary Ca/Cr ratio 0.5	>200 *µ*g/g 135–145 mmol/L 3–5.4 mmol/L 0.66–1.07 mmol/L 0 mg/dL 0.33

11 weeks	*Gastrointestinal findings* First episode of intestinal obstruction Duodenogastric intussusception	Glycemia between 4 and 10 No hypoglycemia	
	*Cardiac findings* Obstructive hypertrophy		
	*Neurological findings* Moderate axial hypotonia		

14 weeks	*Multiorgan impairment* Second episode of intestinal obstruction with cardio-respiratory distress and death	Glycemia 5	

IGF_1_ = insulin-like growth factor 1, IGFBP_3_ = insulin-like growth factor-binding protein 3, GH = growth hormone, ALT = alanine transaminase, AST = aspartate transaminase, Ca = calcium, and Cr = creatinine.

## Data Availability

The data used to support the findings of this case report are included within the article.

## References

[1] Ogilvy-Stuart A. L., Soos M. A., Hands S. J., Anthony M. Y., Dunger D. B., O’Rahilly S. (2001). Hypoglycemia and resistance to ketoacidosis in a subject without functional insulin receptors. *Journal of Clinical Endocrinology and Metabolism*.

[2] Termote J. U. M., Breur J. M., de Vroede M. A. (2016). Hypertrophic cardiomyopathy in Donohue syndrome. *Cardiology in the Young*.

[3] Simpkin A., Cochran E., Cameron F. (2014). Insulin receptor and the kidney: nephrocalcinosis in patients with recessive *INSR* mutations. *Nephron Physiology*.

[4] Musso C., Cochran E., Moran S. A. (2004). Clinical course of genetic diseases of the insulin receptor (type A and Rabson-Mendenhall syndromes): a 30-year prospective. *Medicine*.

[5] Weber D. R., Stanescu D. E., Semple R., Holland C., Magge S. N. (2014). Continuous subcutaneous IGF-1 therapy via insulin pump in a patient with Donohue syndrome. *Journal of Pediatric Endocrinology & Metabolism: Journal of Pediatric Endocrinology & Metabolism*.

[6] Brisigotti M., Fabbretti G., Pesce F. (1993). Congenital bilateral juvenile granulosa cell tumor of the ovary in leprechaunism: a case report. *Fetal and Pediatric Pathology*.

[7] Kawashima Y., Nishimura R., Utsunomiya A. (2013). Leprechaunism (Donohue syndrome): a case bearing novel compound heterozygous mutations in the insulin receptor gene. *Endocrine Journal*.

[8] Kostopoulou E., Shah P., Ahmad N., Semple R., Hussain K. (2017). Gastrointestinal dysmotility and pancreatic insufficiency in 2 siblings with Donohue syndrome. *Pediatric Diabetes*.

[9] Choi G., Je B., Kim Y. J. (2022). Gastrointestinal emergency in neonates and infants: a pictorial essay. *Korean Journal of Radiology*.

[10] Edwards E. A., Pigg N., Courtier J., Zapala M. A., MacKenzie J. D., Phelps A. S. (2017). Intussusception: past, present and future. *Pediatric Radiology*.

[11] Alarcon C., Morales A. V., Pimentel B., Serna J., de Pablo F. (1998). (Pro) insulin and insulin-like growth factor I complementary expression and roles in early development. *Comparative Biochemistry and Physiology Part B: Biochemistry and Molecular Biology*.

[12] Rinderknecht E., Humbel R. E. (1978). The amino acid sequence of human insulin-like growth factor I and its structural homology with proinsulin. *Journal of Biological Chemistry*.

[13] Boucher J., Softic S., El Ouaamari A. (2016). Differential roles of insulin and IGF-1 receptors in adipose tissue development and function. *Diabetes*.

[14] Pires K. M., Buffolo M., Schaaf C. (2017). Activation of IGF-1 Receptors and Akt signaling by systemic hyperinsulinemia contributes to cardiac hypertrophy but does not regulate cardiac autophagy in obese diabetic mice. *Journal of Molecular and Cellular Cardiology*.

[15] Shiomi-Sugaya N., Komatsu K., Wang J. W., Yamashita M., Kikkawa F., Iwase A. (2015). Regulation of secondary follicle growth by theca cells and insulin-like growth factor 1. *Journal of Reproduction and Development*.

[16] Freier S., Eran M., Reinus C. (2005). Relative expression and localization of the insulin-like growth factor system components in the fetal, child and adult intestine. *Journal of Pediatric Gastroenterology and Nutrition*.

[17] Wang J., Niu W., Nikiforov Y. (1997). Targeted overexpression of IGF-I evokes distinct patterns of organ remodeling in smooth muscle cell tissue beds of transgenic mice. *Journal of Clinical Investigation*.

[18] Nijim Y., Awni Y., Adawi A., Bowirrat A. (2016). Classic case report of Donohue syndrome (leprechaunism; OMIM ∗246200): the impact of consanguineous mating. *Medicine*.

[19] Azzabi O., Jilani H., Rejeb I. (2016). Arg924X homozygous mutation in insulin receptor gene in a Tunisian patient with Donohue syndrome. *Journal of Pediatric Endocrinology & Metabolism: Journal of Pediatric Endocrinology & Metabolism*.

[20] Kwon H. J., Park M. I., Park S. J. (2019). Insulin resistance is associated with early gastric cancer: a prospective multicenter case control study. *Gut and Liver*.

